# Identification of miRNAs during mouse postnatal ovarian development and superovulation

**DOI:** 10.1186/s13048-015-0170-2

**Published:** 2015-07-08

**Authors:** Hamid Ali Khan, Yi Zhao, Li Wang, Qian Li, Yu-Ai Du, Yi Dan, Li-Jun Huo

**Affiliations:** Key Laboratory of Agricultural Animal Genetics, Breeding and Reproduction, Education Ministry of China, College of Animal Science and Technology, Huazhong Agricultural University, Wuhan, 430070 People’s Republic of China

**Keywords:** Non-coding RNAs, Deep sequencing, Ovarian development, Mouse

## Abstract

**Background:**

MicroRNAs are small noncoding RNAs that play critical roles in regulation of gene expression in wide array of tissues including the ovary through sequence complementarity at post-transcriptional level. Tight regulation of multitude of genes involved in ovarian development and folliculogenesis could be regulated at transcription level by these miRNAs. Therefore, tissue specific miRNAs identification is considered a key step towards understanding the role of miRNAs in biological processes.

**Methods:**

To investigate the role of microRNAs during ovarian development and folliculogenesis we sequenced eight different libraries using Illumina deep sequencing technology. Different developmental stages were selected to explore miRNAs expression pattern at different stages of gonadal maturation with/without treatment of PMSG/hCG for superovulation.

**Results:**

From massive sequencing reads, clean reads of 16–26 bp were selected for further analysis of differential expression analysis and novel microRNA annotation. Expression analysis of all miRNAs at different developmental stages showed that some miRNAs were present ubiquitously while others were differentially expressed at different stages. Among differentially expressed miRNAs we reported 61 miRNAs with a fold change of more than 2 at different developmental stages among all libraries. Among the up-regulated miRNAs, mmu-mir-1298 had the highest fold change with 4.025 while mmu-mir-150 was down-regulated more than 3 fold. Furthermore, we found 2659 target genes for 20 differentially expressed microRNAs using seven different target predictions programs (DIANA-mT, miRanda, miRDB, miRWalk, RNAhybrid, PICTAR5, TargetScan). Analysis of the predicted targets showed certain ovary specific genes targeted by single or multiple microRNAs. Furthermore, pathway annotation and Gene ontology showed involvement of these microRNAs in basic cellular process.

**Conclusions:**

These results suggest the presence of different miRNAs at different stages of ovarian development and superovulation. Potential role of these microRNAs was elucidated using bioinformatics tools in regulation of different pathways, biological functions and cellular components underlying ovarian development and superovulation. These results provide a framework for extended analysis of miRNAs and their roles during ovarian development and superovulation. Furthermore, this study provides a base for characterization of individual miRNAs to discover their role in ovarian development and female fertility.

**Electronic supplementary material:**

The online version of this article (doi:10.1186/s13048-015-0170-2) contains supplementary material, which is available to authorized users.

## Background

Ovarian folliculogenesis is a complex biological process, which is tightly regulated by the coordination of large number of genes [1]. In animals, developmental process starts with oogenesis when RNA and protein are combined resulting in the growth of oocyte. In addition, oocyte development is also regulated by complex genetic network especially transcription regulators [2]. The extent of transcription reflects the importance of messenger RNA (mRNA) during the growth of oocytes, hence early development of oocyte is exclusively dependent on the maternally inherited components, including proteins and RNAs [3]. So far, advanced technology led to the discovery of some non-coding RNAs like small nucleolar RNAs, small interfering RNAs, microRNAs and antisense RNAs, thus suggesting that eukaryotic transcriptome is much more complex than expected [4]. MicroRNAs (miRNAs) belongs to small non-coding RNAs which are of prime importance due to their roles in regulating genes and genomes at different levels such as chromatin structure, chromosome segregation, transcription and RNA processing [5]. Likewise mRNA, microRNA expression shows vibrant changes during the development process as extensive number of genes involved in the process of oogenesis, are influenced by miRNAs [6].

miRNAs are miniature (typically ~22 nucleotides in length) non-coding RNAs that play significant roles in post-transcriptional regulation of specific mRNAs. Most miRNAs arise from very long transcripts known as primary miRNA (pri-miRNA) by drosha and its cofactor DGCR8 (DiGeorge syndrome critical region gene 8) in nucleus converting it to ~70-100 bp precursor miRNA (pre-miRNA). After the transport of pre-miRNAs from nucleus to cytosol, Dicer (a RNA III endonuclease) process precursor miRNA by removing hairpin loop thus converting it to mature miRNA [7]. Previous studies suggested that conditional knockout of Dicer in the ovary leads to sterility; thus providing strong evidence of miRNAs involvement in ovarian development [8]. Furthermore, Amhr2-Cre mediated deletion of Dicer in mice resulted in reduced ovarian function due to loss of miRNAs [9–11]. Dicer1 conditional knockout (cKO) mice shows accelerated early follicles recruitment and more degenerate follicles in ovaries. Furthermore, significant differences were noted in some follicle development related genes suggesting that miRNA expression is time and gene dependent [12].

miRNA and mRNA interactions through direct base-pairing causes suppression of translation or assist mRNA degradation in sequence specific manner [13, 14]. By this way miRNAs influence various cellular processes e.g*.,* development, cell proliferation and differentiation, self-renewal and apoptosis etc. [14]. Also, the mechanism of miRNA mediated gene regulation is quite complex, as a single miRNA can target thousands of genes transcripts and vice versa [15]. Recent studies have shown that certain reproductive processes are strictly regulated at the transcriptional and post-transcriptional levels [16]. Along with, a novel mechanism of miRNA mediated post-transcriptional regulation has revealed lately which is regarded as an important regulator of reproductive processes [17, 18].

Folliculogenesis is a complex process involving series of morphological and functional changes depending on the type of cells and developmental stage [19]. Previous investigations have evaluated miRNA transcriptomes from the reproductive organs in different organisms to decipher their expression profile and have shown their roles in pathology, fertility and development of ovary [14, 16, 20–22]. Although these findings provide valuable information about individual miRNAs differentially expressed in specific type of ovarian cells with/without response to gonadotropic hormones, the number of experimentally validated miRNAs expressed in the ovary is still very limited. For example, miR-132 and miR-212 respond to luteinizing hormone (LH)/human chorionic gonadotropin (hCG) thus, these miRNAs play important roles in post-transcriptional regulation of granulosa cells [23]. Similarly, miR-224, miR-21 and miR-145 regulate proliferation and apoptosis of granulosa cells [24–26].

Prior cloning and sequencing techniques identified different number of miRNAs at specific stage of ovarian development. For example, Ro et al. identified 122 miRNAs from adult mice ovary while 516 miRNAs were identified from new born mice ovary by Ahn et al. [23, 27]. Mishima et al. and Tripurani et al. reported expression of 154 miRNAs and 58 miRNAs in adult mice ovary and bovine fetal ovary, respectively [1, 28]. However, these studies provide limited information about involvement of miRNAs in postnatal development. Therefore, identifying the expression pattern of miRNAs in mouse ovary at different stages of ovarian development became the key step to discover their roles in ovarian development and folliculogenesis.

To date, number of experimentally validated miRNAs playing vital roles in ovarian development is quite insufficient. Thus, the exceptional volume of sequence data generated from our work provided distinctive opportunity to mine for differentially expressed as well as novel miRNAs that have evaded previous cloning and sequencing techniques. This data is in line with expression pattern of experimentally validated miRNAs implying the authenticity of the differentially expressed miRNAs in this study. Furthermore, we investigated potential novel miRNAs along with differentially expressed miRNAs and predicted their roles in various pathways and Gene ontologies (GOs). Moreover, this study provided important information about the miRNAs expression pattern during postnatal development and superovulation in female mice. This further provides baseline for experimental validation of these differentially expressed and potential novel miRNAs to reveal their respective roles and regulatory mechanism during postnatal development and ovulation process at the molecular level.

## Methods

### Animal treatment

Kunming female mice were obtained from the Centre of Laboratory Animals of Hubei Province (Wuhan, PR China). Mice were housed under controlled temperature (20 °C −24 °C) and lighting (12 h light/12 h darkness) with food and water *ad libitum*. All animal treatment procedures were approved by the Ethical Committee of the Hubei Research Center of Experimental Animals (Approval ID: SCXK (Hubei) 2008–0005).

Primordial follicle activation are known to occur and begin to develop in the ovary of 3 days old female mice, and 21 days old female mice at stage of puberty begin to ovulate for the first time. Furthermore, in the preliminary experiment, we found that most follicles in 6 days old, 8 days old, 12 days old and 15 days old mice ovaries are primary follicles, secondary follicles with 2–3 layers of granulosa cells, and secondary follicles with multiple layers of granulosa cells, respectively. Therefore, we obtained ovaries from 6 days old (6d), 8 days old (8d), 12 days old (12d), 15 days old (15d) and 21 days old (21d) of Kunming white female mouse for analysis of microRNAs expression profile during postnatal development and follicular development after primordial follicle activation. For analysis of microRNAs expression during ovulation, 21d old mice were injected with 10 IU of pregnant mare serum gonadotropin (PMSG) for 48 h and then with 10 IU of human chorionic gonadotropin (hCG). Mice were scarified by cervical dislocation and ovaries were collected at 6 h and 48 h after PMSG and 6 h after hCG treatment and RNA was extracted for deep sequencing of miRNAs expression profile to reveal the response of miRNAs to PMSG/hCG and during super-ovulation. Therefore, the ovary samples were marked as 6d, 8d, 12d, 15d, 21d, P6 (PMSG 6 h), P48 (PMSG 48 h), and h6 (PMSG 48 h and hCG 6 h). For each library preparation, total RNA was pooled isolated from ovaries of at least 10 female mice.

### Small RNA library construction and deep sequencing

Total RNA was extracted from ovaries using Trizol reagent (Invitrogen, Carlsbad, CA, USA) following manufacturer protocol and RNA quality was analyzed by using nanodrop ND-8000 spectrophotometer (Thermo Electron Corporation, USA) at 260/280 nm. From each sample, 2 μg of total RNA was used for deep sequencing using Hiseq 2000 sequencing platform from illumina (Illumina, San Diego, CA, USA) at Genergy Biotechnology Co., Ltd., Shanghai, China). Briefly, 16 to 26 nt small RNA fraction was purified from total RNA and enriched from denaturating polyacrylamide gel electrophoresis (PAGE). Adapters were ligated at 3’ and 5’ ends using T4 ligase and further small RNA was subjected to RT-PCR for amplification (12 Cycles). PCR product was further purified using polyacrylamide TBE (Tris/Borate/EDTA) gel and used for sequencing. Sequencing files were extracted from image file generated by Illumina genome analyzer.

### Bioinformatics analysis and statistics

After filtering out adapters sequences and low quality reads, clean reads were mapped to UCSC mouse genome mm9 (http://genome.ucsc.edu/) using NCBI Mega BLAST. Moreover Rfam version 10.1 (http://rfam.sanger.ac.uk/) was used for removal of other non-coding RNAs. Remaining sequences were analyzed for miRNAs using BLAST search against miRNA database (miRBase V.20, www.mirbase.org) to identify conserved microRNAs in mouse (*Mus musculus*). Perfectly matched sequences were regarded as conserved sequences.

### Differential expression analysis

To analyze differentially expressed microRNAs from all eight libraries (6d, 8d, 12d, 15d, 21d, P6, P48 and h6), we used the criteria as reported by others. Briefly, miRNA expression was normalized to get the expression of transcript per million by using the formula. Normalized expression = (Actual miRNA sequencing reads count/Total clean reads count) × 1,000,000. After normalization, the expression values of non-detected miRNAs were revised to 0.01. miRNAs whose normalized expression value was <1 in both samples [e.g., in case of 6d-8d, 6d is (sample 1) while 8d (sample 2)] were excluded from the following differential expression analysis due to low expression. Statistical significance of miRNA expression in each group was calculated using Bioconductor R package [29–33].

### Quantitative RT- PCR (qRT-PCR)

To validate the differentially expressed miRNAs identified using deep sequencing technology, eight miRNAs were further selected and their relative expression levels were analyzed in different sized follicles (i.e., 100 μm −130 μm, 200 μm -280 μm, 450 μm -550 μm, 500 μm -600 μm isolated from ovary samples of 12d, 21d, P48, and h6, the same as in sequencing samples respectively). miRNA was extracted using miRcute miRNA Isolation Kit (Tiangen, Beijing China) according to manufacturer protocol. cDNA was synthesized using miScript II RT Kit (QIAGEN) and qRT-PCR was performed using the miScript SYBR Green PCR Kit **(**QIAGEN**)** according to the manufacturer’s protocol. The reaction mixtures were incubated in a 96-well plate at 95 °C for 15 min followed by 40 cycles of 94 °C for 15 s, 60 °C for 30 s and 70 °C for 30 s. All reactions were run in triplicate. The primers for miRNAs have the same sequences as *Mus Muscullus* miRNAs with an appropriate adjustment at their 5’ terminus. Expression of target miRNA in each sample was normalized to the small nuclear gene U6. Relative miRNA levels were calculated using the comparative threshold 2^−ΔΔCt^ method [34].

### Statistical analysis

RNA-seq data is presented as means ± standard deviations (SD). Differences between samples were regarded as significant at p < 0.01. Furthermore, each miRNA expression level is presented as 2^−ΔΔCt^ means ± SE (standard error), and error bars indicate the standard error of 2^−ΔΔCt^ mean values. To examine the significance of differential expression level in each miRNA between different size follicles One-way ANOVA and Duncan’s Multiple Range test were used by using SPSS (Version17.0; SPSS, Chicago, IL, USA). The difference was considered as significant when P <0.05.

## Results

### Sequence analysis of small RNAs in mouse ovary

**Fig. 1:**
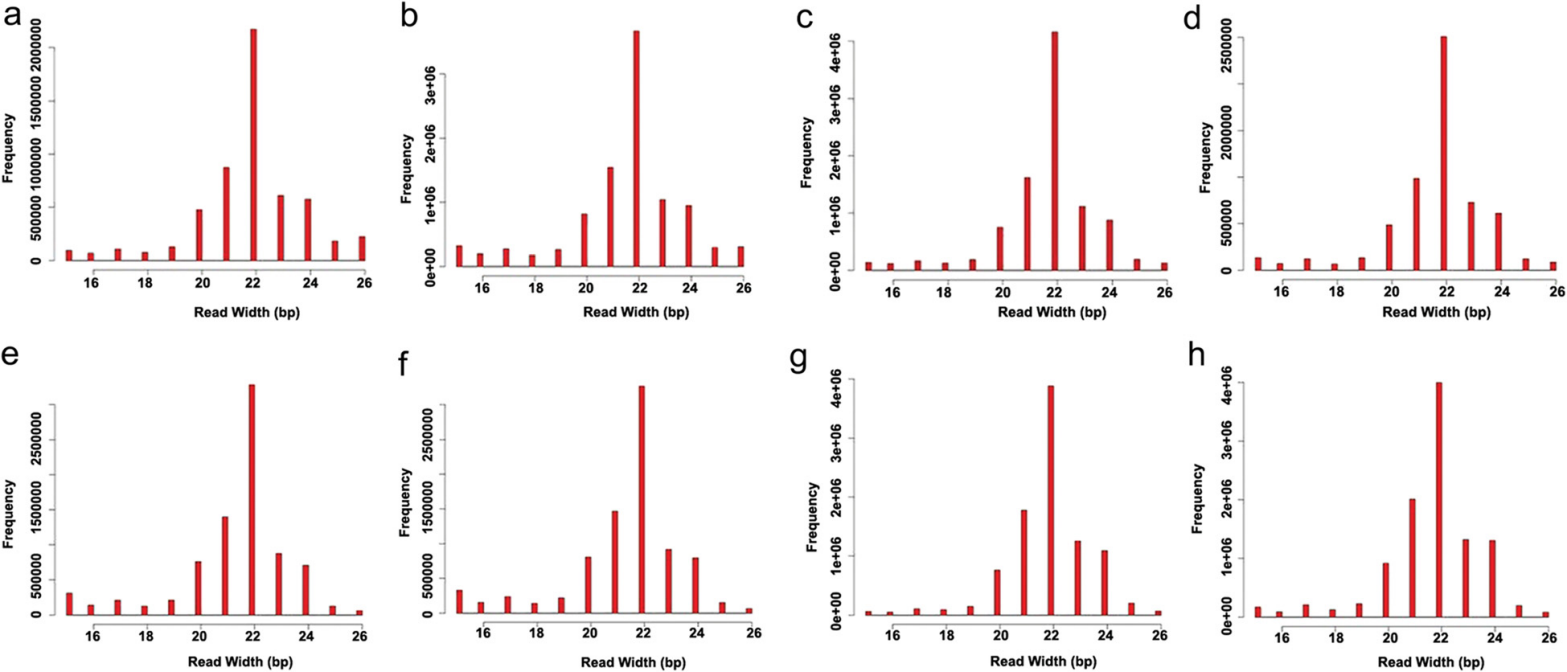
Length distribution and abundance of miRNA sequences in mouse ovary by Illumina deep sequencing. Sequence length distribution of clean reads based on the abundance and distinct sequences; the most abundant size class was 22 nt, followed by 21 nt and 23 nt. **a**) 6d, **b**) 8d, **c**) 12d, **d**) 15d, **e**) 21d, **f**) P6 **g**) P48, **h**) h6

To investigate miRNAs involved in the postnatal development and ovulation of mouse ovary, eight small RNA libraries were constructed by Illumina Hiseq 2000 small RNA deep sequencing technology. Raw reads were processed by filtering out low quality sequences, empty adapters and single read sequences. Clean reads of 16–26 nt (Fig. 1) were selected for further analysis from mice postnatal development and superovulated sequenced libraries, respectively (Table 1).

**Table 1:** Number of reads of small RNA libraries from mice ovaries

S.No	Sample	Sample Id	Clean Data (Read Num)	Reads (>=1 alignment)	Reads failed to align
1.				5223449	353537
	Day 6	6d	5576986	(93.66 %)	(6.34 %)
2.				9068323	770439
	Day 8	8d	9838762	(92.17 %)	(7.83 %)
3.				9078152	499455
	Day 12	12d	9577607	(94.79 %)	(5.21 %)
4.				5647332	417152
	Day 15	15d	6064484	(93.12 %)	(6.88 %)
5.				7524492	701170
	Day 21	21d	8225662	(91.48 %)	(8.52 %)
6.				7829348	723139
	PMSG 6 h	P6	8552487	(91.54 %)	(8.46 %)
7.				9019698	487799
	PMSG 48 h	P48	9507497	(94.87 %)	(5.13 %)
8.				9947594	707958
	PMSG-48 h +hCG 6 h	h6	10655552	(93.36 %)	(6.64 %)

The source is UCSC genome database mm9. Clean data refers to removal of adopters and low quality reads

### Differentially expressed miRNAs during postnatal development and superovulation in mouse ovaries

**Fig. 2:**
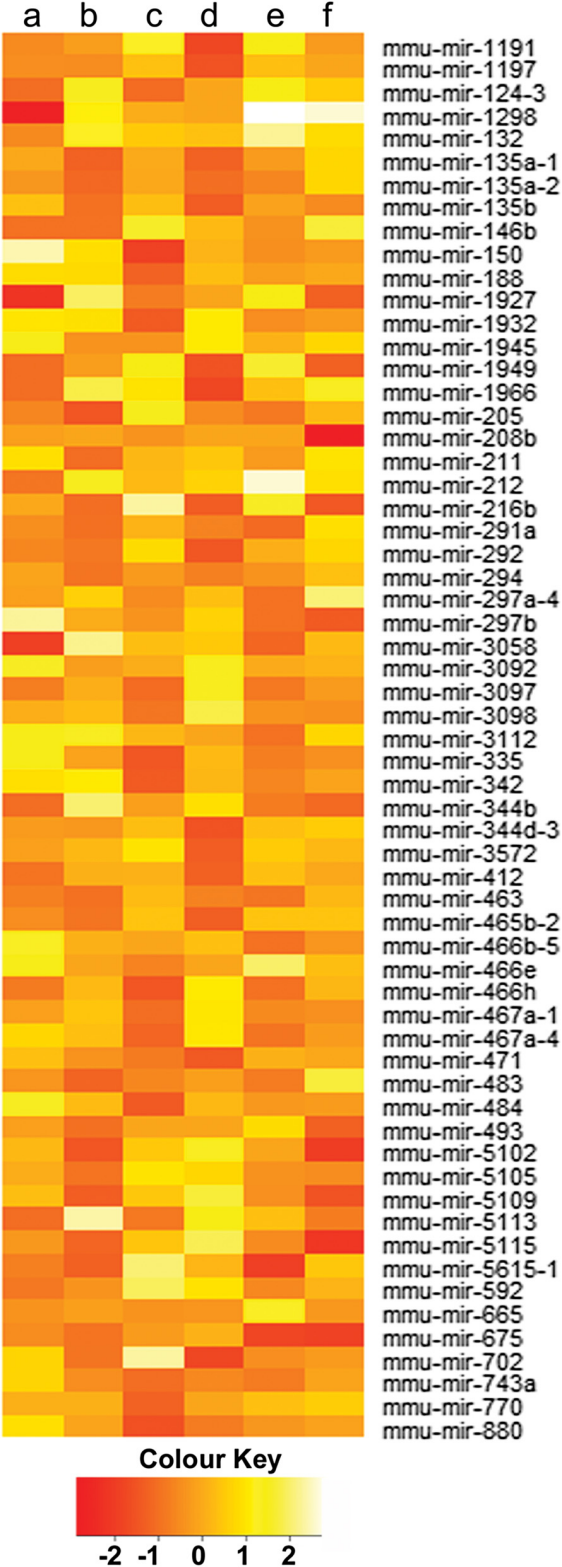
Heatmap showing differentially expressed miRNAs. Heat map shows differentially expressed miRNAs among six libraries with a log_2_Ratio ≥ 1. Bright red and light yellow represent down-regulated and up-regulated miRNAs respectively. **a**) 6d-8d, **b**) 8d-12d, **c**) 12d-15d, **d**) 15d-21d, **e**) 21d-P6, **f**) P48-h6

The main purpose of this study was to identify miRNAs involved in mouse ovarian development and folliculogenesis. According to the changes in relative miRNA expression among eight libraries representing postnatal developmental and superovulated ovaries, in total 58, 73, 64, 31, 24, 21 miRNAs were differentially expressed during 6d-8d, 8d-12d, 12d-15d, 15d-21d, 21d-P6, P48-h6 respectively (|log_2_Ratio| ≥ 1, *P*-value ≤ 0.01). Further analysis showed that among all differentially expressed miRNAs, 61 miRNAs showed more than two fold differences in terms of expression. Among the up-regulated miRNAs, mmu-mir-1298 had the highest fold change with 4.025 during 21d-P6 followed by mmu-mir-212 and mmu-mir-132 with a fold change of 3.71 and 3.28, respectively. Among down regulated miRNAs, mmu-mir-150 was down-regulated more than 3 fold during 12d-15d (Fig. 2).

### qRT-PCR analysis of miRNAs expression in ovarian follicles

**Fig. 3:**
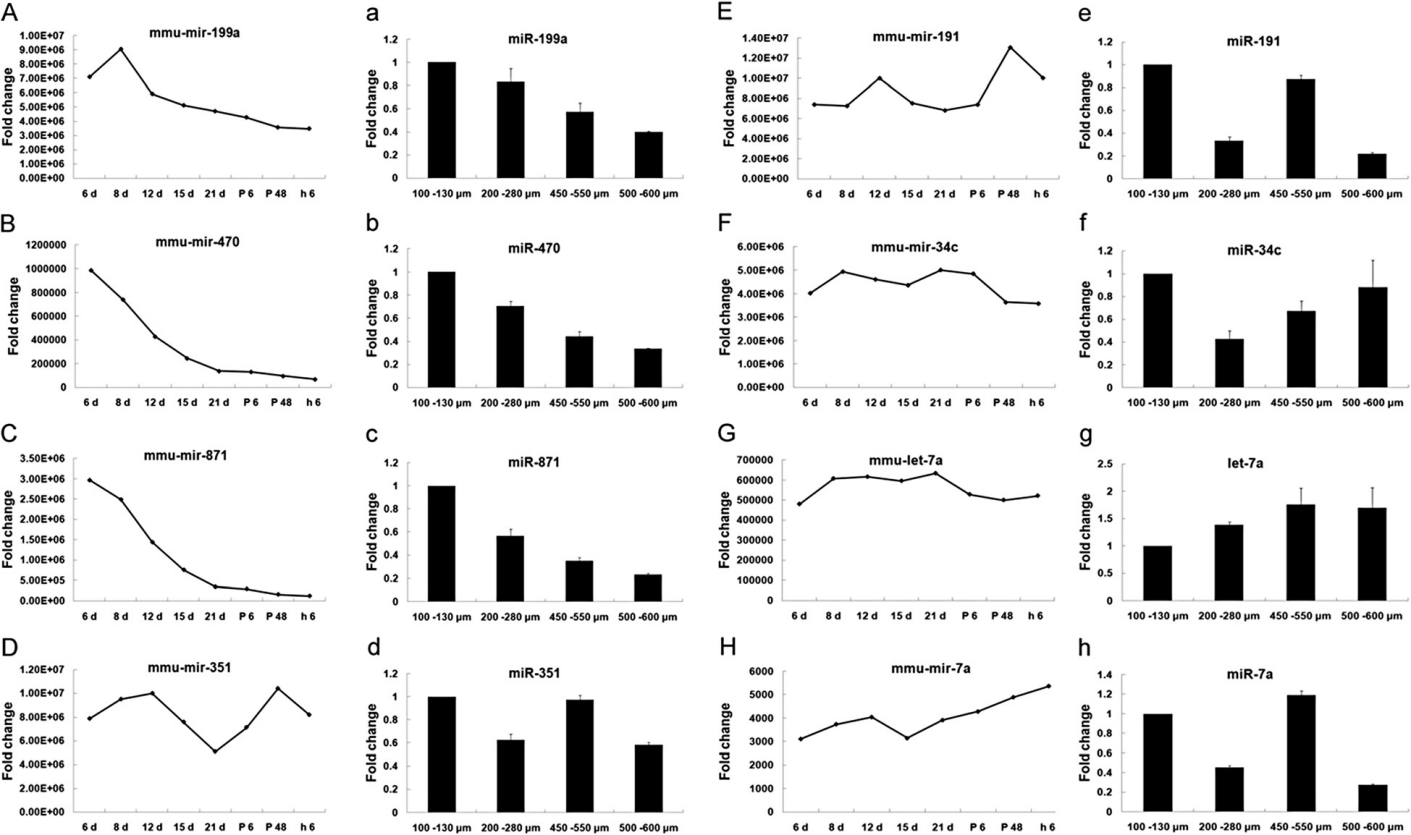
qRT-PCR validation of eight miRNAs in different sized follicle. qRT–PCR of selected known miRNAs in different size follicles (100 μm −130 μm, 200 μm -280 μm, 450 μm -550 μm, 500 μm -600 μm). miRNA was isolated and cDNA was synthesized from different size follicles followed by qRT-PCR. Capital letters shows expression profile of respective miRNA in deep sequencing data while small letters represents their expression profile in different size follicles using qRT-PCR. **A**) Expression profile of mmu-mir-199a in sequencing data. **a**) Expression profile of miR-199a through qRT-PCR. **B**) Expression profile of mmu-mir-470 in sequencing data. **b**) Expression profile of miR-470 through qRT-PCR. **C**) Expression profile of mmu-mir-871in sequencing data. **c**) Expression profile of miR-871 through qRT-PCR. **D**) Expression profile of mmu-mir-351 in sequencing data. **d**) Expression profile of miR-351 through qRT-PCR. **E**) Expression profile of mmu-mir-191 in sequencing data. **e**) Expression profile of miR-191 through qRT-PCR. **F**) Expression profile of mmu-mir-34c in sequencing data. **f**) Expression profile of miR-34c through qRT-PCR. **G**) Expression profile of mmu-let-7a in sequencing data. **g**) Expression profile of let-7a through qRT-PCR. **H**) Expression profile of mmu-mir-7a in sequencing data. **h**) Expression profile of miR-7a through qRT-PCR

To further validate these differentially expressed miRNAs identified from the mouse ovary, the expression levels of miR-199a, miR-470, miR-871, miR-34c let-7a, miR-7a, miR-351, miR-191 were further examined in different size follicles (i.e., 100 μm −130 μm, 200 μm -280 μm, 450 μm -550 μm, 500 μm -600 μm) using qRT-PCR assay. qRT-PCR results showed that some of the miRNAs exhibit developmental stage-specific expression patterns in ovarian development. The expression patterns of miR-199a, miR-470, miR-871, shows relatively higher expression in small preantral follicles as compared to large antral follicles with the similar expression pattern as the results of deep sequencing. However, the expression dynamics of miR-34c, let-7a, miR-7a were different; the expression level was increased with increase in size of follicles. The results indicate that the expression pattern of some microRNAs are consistent with our deep sequencing results (Fig. 3), but others not. Further QRT-PCR assay is needed to validate the expression pattern of selected microRNAs obtained from deep sequencing results.

### Novel miRNAs

Sequencing data was subjected to Rfam to filter out rRNAs, tRNAs, snRNAs and snoRNAs. The processed data was used for novel microRNA identification by miRDeep2, an algorithm based on microRNA biogenesis. miRDeep2 predicted 160 potential novel miRNAs at the relatively stringent score cut-off of 5 and signal-to-noise ratio of 12.1 (Additional file 1: Table S1). For each set of newly identified miRNA, we used a variety of assessment methods to evaluate the predictive accuracy. We used both, False Positive rate (FPR) and True Positive Rate (TPR) for assessment of predicted results. Furthermore, RNA-fold was used to confirm the structure of predicted miRNAs [35]. After filtering out the predicted novel miRNAs by removal of loci matching other RNA genes, keeping only novel miRNAs with significant rand fold *p*-value (<0.05), with miRDeep2 score >5, and analyzing the hairpin structure of the microRNAs, the list was reduced to 10 potential novel microRNAs (Additional file 2: Table S2). For detection of miRNAs in deep sequencing data by miRDeep2, a score cutoff equivalent to a prediction signal-to-noise ratio of 10 is most often used [36].

### Putative target genes of differentially expressed MicroRNA

miRNA mediated gene expression regulation plays significant role in development, maturation and ovulation by governing self-renewal, proliferation, differentiation and apoptosis [37, 38]. To figure out miRNA putative target genes associated with maturation and superovulation of ovary at different stages of development, miRanda public database was used. Target genes of differentially expressed miRNAs were predicted according to previously established criteria [39–42]. For rigorous screening of highly credible miRNA target genes, three basic criteria were used 1) Conservation, 2) Energy, 3) mirSVR score. On the basis of these criteria we selected 71 differentially expressed microRNAs targeting 3324 putative target genes (Data not shown). Additionally, we extended our approach and selected 20 microRNAs from these 71 differentially expressed microRNAs for further validation using seven different target prediction programs (DIANA-mT, miRanda, miRDB, miRWalk, RNAhybrid, PICTAR5, TargetScan) to enhance the credibility of the target genes [43]. Genes targeted by five or more different programs are shown in Additional file 3: Table S3.

**Fig. 4:**
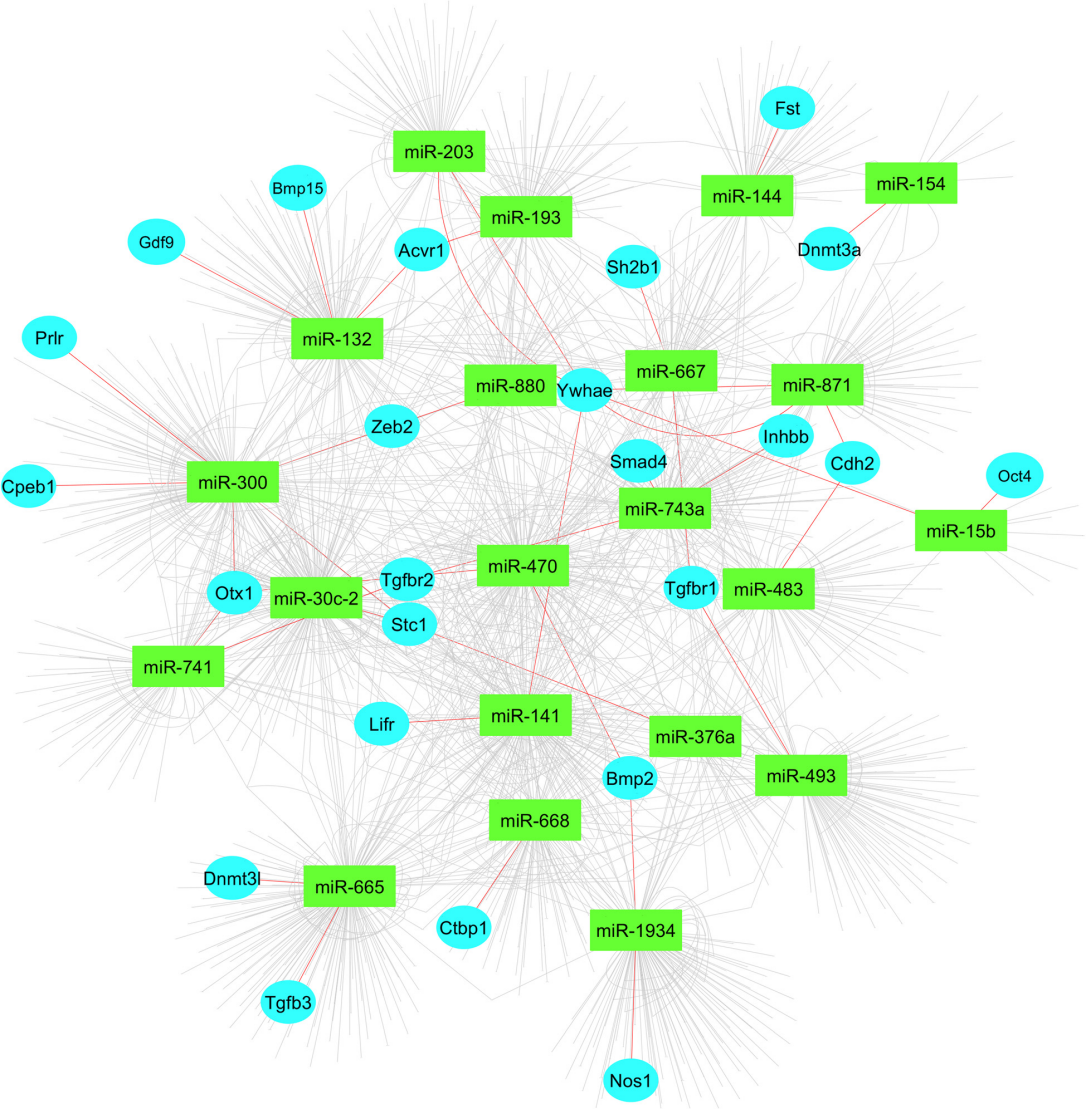
Ovary specific genes targeted by microRNAs. Network shows microRNAs and their predicted target genes. Ovary specific genes are highlighted in the network. Green rectangles represent microRNAs while blue circles represent target genes. The specific interaction is highlighted by red lines between microRNAs and ovary specific target genes

Moreover, among these putative target genes we further identified ovary specific genes. These genes play vital role in development of ovary, folliculogenesis, ovulation and thus influencing female fertility. Changes in miRNA expression pattern regulate these potential target genes hence suggesting a collaborative role between microRNAs and mRNAs during ovarian development. Some of these genes are targeted by multiple microRNAs while in other cases multiple genes are targeted by a single miRNA as shown in Fig. 4.

### Gene ontology and pathway annotation

**Fig. 5:**
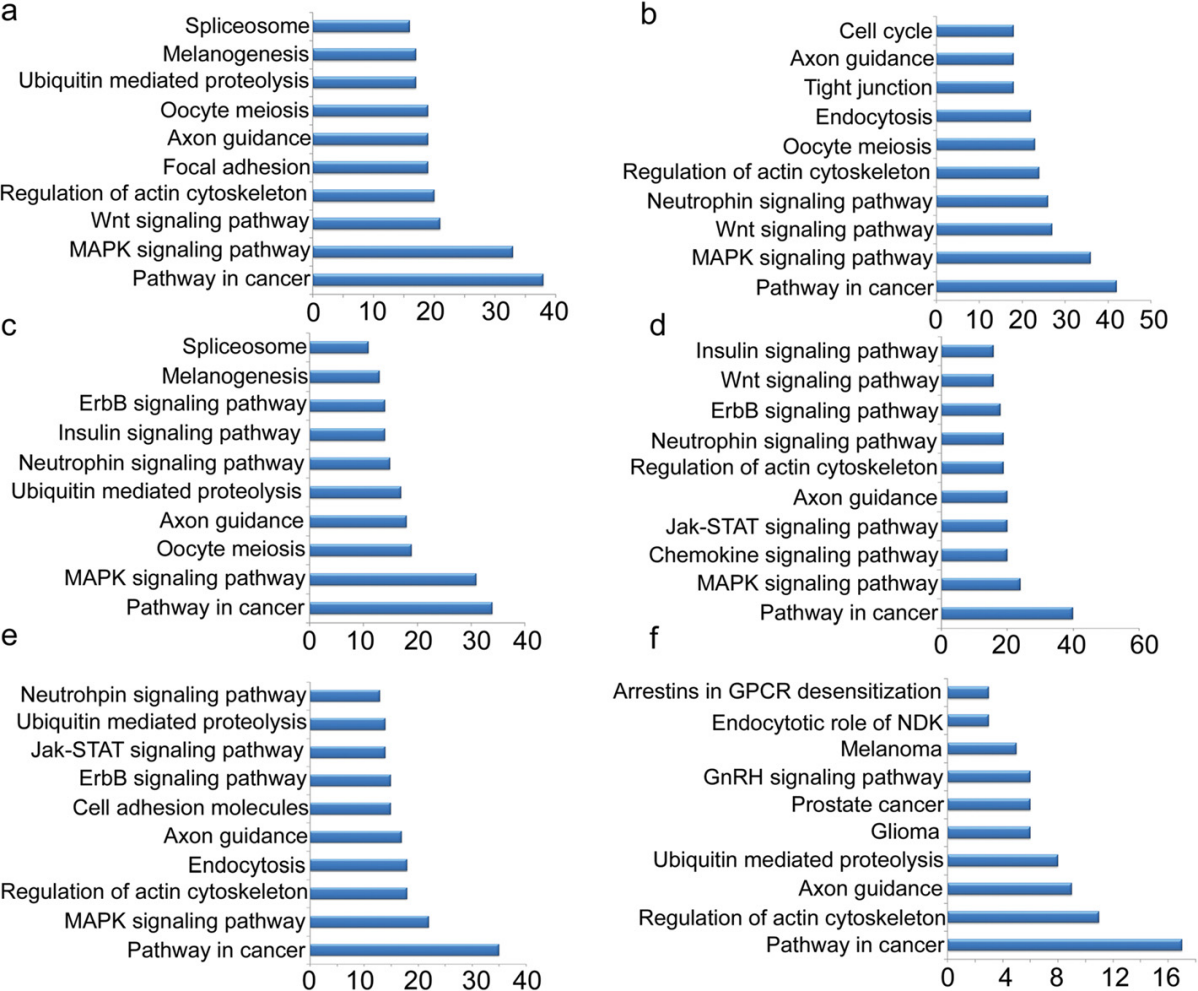
Pathway annotation. Pathway annotation of differentially expressed miRNAs based on predicted target genes involved in different pathways during 6d-8d (**a**), 8d-12d (**b**), 12d-15d (**c**), 15d-21d (**d**), 21d-P6 (**e**), P48-h6 (**f**). Vertical axis shows pathways while horizontal axis shows number of genes involved in respective pathway

For further understanding the roles of differentially expressed miRNAs in physiological functions and biological processes during postnatal development and ovulation, target prediction was performed by using public database (miRanda). Human Gene Ontology (GO) database and Koyoto Encyclopedia of Genes and Genomes (KEGG) database were used for GO annotation and KEGG pathway analysis to identify functional modules regulated by these miRNAs. The GO annotation enrichment results showed that regulation of transcription and regulation of RNA metabolic process were significantly enriched during all the six differentially expressed libraries except P48-h6 in terms of biological function. While regulation of transcription and phosphate metabolic process were among highly enriched biological functions based on number of genes involved during P48-h6 (Additional file 4: Figure S1). Similarly, plasma membrane part and ion binding were among the most significant cellular components and molecular functions in terms of GO annotation (Additional file 5: Figure S2 and Additional file 6: Figure S3). Moreover, KEGG and Biocarta pathway analysis revealed that Pathway in cancer, MAPK signaling pathway, Wnt-signaling pathway and oocyte meiosis ranked among the most enriched pathways (Fig. 5). Although the false-positive predictions always exist, we suggest that these targets have high possibility of being regulated by miRNAs which are involved in the development of mouse ovary and ovulation.

## Discussion

The discovery of miRNAs revolutionized the unanticipated regulation of transcriptome and proteomes. Illumina deep sequencing transformed discovery of miRNAs as this technique is considered an efficient way for miRNA discovery and is widely used to produce small RNA profiles in various organisms. Although some miRNAs have been proved critically involved in the regulation of ovarian granulosa cells by using real time PCR and other techniques, granulosa cells are only one type of cells in follicles while follicles grow inside the ovary and ovary grows as a whole organ during postnatal development. Furthermore, due to the complexity of ovarian development and folliculogenesis, the study of single or multiple miRNAs only in granulosa cells might have some limits, which could not reflect the changes in profile of miRNAs and the regulation of target genes involved in ovarian development and folliculogenesis. Herein, detailed miRNA profiles of mice ovaries at 6d, 8d, 12d, 15d, 21d, P6, P48 and h6 using Illumina deep sequencing technique were obtained in this study. These results reported the miRNA expression profiles at different time points of postnatal development and superovulation from mice ovaries, which at least partially represent the different stages of folliculogenesis. Furthermore, the differentially expressed miRNAs and their target genes were also revealed between the near groups, which could efficiently reflect the dynamic changes of miRNAs during ovarian development and folliculogenesis. The gene ontology and pathway annotation of target genes of those differentially expressed miRNAs were further analyzed to reveal the dynamic changes of biological and cellular processes inside of the ovary during postnatal development and ovulation. We suggest that present work provides important information for understanding the biological and cellular processes and regulation of miRNA and target genes in the whole ovary during postnatal maturation and folliculogenesis.

In present study, the sequencing analysis showed that the dominant size of small RNAs in mice ovary was 22 nt followed by 21 and 23 nt sequences (Fig. 1). These results resemble to typical Dicer-processed small RNA products with known 19–24 nt range for miRNAs. Our sequencing data is consistent with previous findings in mice [28] and pig [44], but vary from Holstein Cattle ovary where the 20 nt size was the most abundant, followed by 22 nt [45]. Another study in bovine ovary indicated that 21 nt is the predominant size [37], possibly because of difference in species.

In liberaries from postnatal developmental and superovulated mice ovaries, let-7 miRNA family was abundantly cluster with let-7a being the most abundantly expressed miRNA. Previous finding also showed abundant expression of let-7 miRNA family in the ovary and oocyte of bovines [1, 45, 46], as well as in murine ovaries and testis [47]. Thus, relative abundance suggests that members of let-7 family have important roles in cell fate determination and associated with regulating housekeeping genes during ovarian development [48]. Furthermore, mmu-mir-101, mmu-mir-148a, mmu-mir-26a, and mmu-mir-30d were profuse in our sequencing libraries, as already reported in other animal gonads [1, 28, 37].

Likewise, mmu-mir-21, mmu-mir-125b, mmu-mir-16b, mmu-mir-143 and mmu-mir-199a-3p were expressed abundantly in all libraries despite of changes in expression with development thus suggesting its role in basic reproductive activities. These miRNAs were also reported previously to be among the most prevalent miRNAs in whole ovaries of mice, cattle and pigs [28, 37, 44–46, 49]. Others predominantly expressed miRNAs e.g., mmu-mir-125b, mmu-mir-199a-3p, mmu-mir-29a and mmu-mir-15b targets several ovarian genes and involved in several biological functions like cell signaling, cell death, cell cycle regulation, cellular growth and differentiation and endocrine system [37]. During superovulation, mmu-mir-351, mmu-mir-30c, mmu-miR-26a, mmu-mir-25 expressed extensively as already reported by Fiedler et al. using microarray technology [50]. High expression of mmu-mir-322 shows its involvement in cell differentiation, folliculogenesis and overall ovarian development [51]. Therefore, these miRNAs and their target genes are greatly associated with basic ovarian functions and cellular processes.

Previous studies reported that up-regulation of miR-21 in murine granulosa cells pre and post hCG/LH surge arresting apoptosis in preovulatory granulosa cells. In addition, increased apoptosis and reduced ovulation rate was observed in granulosa cells with knockdown of miR-21 [25, 50]. In current study, differential expression of mmu-mir-21 exhibited significant fold change i.e., 1.34-fold during 21d-P6, even more significant response to hCG, suggesting that previous findings are in concordance with our deep sequencing results. Likewise, Guijun et al. reported that miR-145 suppressed mouse granulosa cells proliferation by targeting ACR1B via activin induced SMAD2 phosphorylation [26]. Differential analysis of mmu-mir-145 showed down-regulation with ovarian growth i.e., log_2_ fold change was 1.53 during 6d-8d and −1.12 during 12d-15d thus showing its roles in cell proliferation.

miRanda algorithm showed that, activin receptor 1 (ACVR1) is predicted target gene for mmu-mir-193, mmu-mir-294, mmu-mir-295 and mmu-mir132. ACVR1 mRNA is present in granulosa-luteal cells and cumulus oocyte complexes during in vitro maturation which play roles in follicular development and steroid metabolism [52, 53]. Bioinformatics analysis showed that mmu-mir-470 targets TGIF1 (TGFB-induced factor homeobox 1) while mmu-mir-300 and mmu-mir-880 targets ZEB2 (zinc finger E-box binding homeobox 2), showing participation in the regulation of TGF-β signaling [54]. As TGF-β signaling is essential for folliculogenesis and oogenesis in mammalian ovaries [55], hence implied the indirect involvement of these miRNAs in folliculogenesis and oogenesis. Furthermore, miR-124 is reported to be actively involved in the suppression of SOX9 which is testis development gene, to inhibit production of SOX9 protein in ovary [56].

Experimental validation of miRNA targets is a challenging approach which ultimately led to the use of in *silico* approaches to predict miRNAs targets [57]. Until now, many algorithms have been designed based on different pairing approaches between miRNA and mRNA [57]. In current study, we used miRanda algorithm for target gene prediction which was initially designed for the fruit fly and then extended to other organisms including mouse. miRanda algorithm is mainly based on energy involved between miRNA:mRNA physical interaction [58]. To further ascertain the miRNA target interaction we used seven different target prediction programs for differentially expressed microRNAs. We identified many putative genes targeted by differentially expressed miRNAs involved in the postnatal maturation and ovulation in mouse. Some of these predicted target genes play key roles in gonadal maturation and ovulation (Fig. 4). For example, TGF-β superfamily members are involved granulosa cell proliferation, estrogens, and progesterone production [59]. Inhibin and activin play significant roles in follicular development and differentiation [60]. Receptors for BMPs (Bone morphogenetic proteins) are present in ovaries, thus play role in differentiation of granulosa cells [61].

Due to challenges in experimental validation of miRNAs targets, *in silico* tools are better approach for target prediction based on different base pairing properties between miRNA and mRNA [62]. The better approach is to use several target prediction tools and due to this reason we used this approach for some differentially expressed microRNAs. Taken together, our findings and other evidences support that these differentially expressed miRNAs play key role in ovarian development and fertility. Analyzed target genes shows involvement in broad range of signaling cascades and pathways of the ovarian function.

The above findings as well as our qRT-PCR results of individual miRNAs are consistent with our deep sequencing data implying high significance of our data and suggesting the critical roles of these differentially expressed miRNAs not experimentally validated so far in ovarian development and folliculogenesis. Further studies will be needed to validate the biological significance of these differentially expressed and novel miRNAs identified in present work, to reveal its specific roles and regulatory mechanism in specific cells of ovary during postnatal development and ovulation.

## Conclusions

This study explored and evaluated microRNA transcriptome in mouse postnatal ovarian development and superovulation at different stages, thus provided valuable information about the dynamic changes of miRNAs profile during ovarian development. Results shows that some of microRNAs either up- or down-regulated during specific period thus indicating their role at a specific stage of ovarian development. Moreover, predicted target genes showed involvement in different pathways and GO terms. Along with, we also reported 10 novel miRNAs that evaded previous sequencing techniques. Further functional characterization of these differentially expressed and novel microRNAs at specific stage of ovarian development will help to elucidate their specific role in follicle growth, ovarian development as well as ovulation. The information we provided in present study will help to identify candidate miRNAs targeting specific molecular and cellular pathways important for follicular development, ovulation as well as ovarian dysfunction.

## Additional files

Additional file 1: Table S1. Survey of miRDeep2 performance for score cut-offs 0 to 10. Additional file 2: Table S2. List of putative novel microRNAs. Additional file 3: Table S3. List of putative target genes. Additional file 4: Figure S1. Partial GO enrichment in biological process. The figure shows partial gene enrichment for differentially expressed miRNAs in terms of biological process during 6d-8d (a), 8d-12d (b), 12d-15d (c), 15d-21d (d), 21d-P6 (e), P48-h6 (f). Vertical axis shows GO terms while horizontal axis shows number of genes involved. Additional file 5: Figure S2. Partial GO enrichment in cellular components. The figure shows partial gene enrichment for differentially expressed miRNAs in terms of cellular components during 6d-8d (a), 8d-12d (b), 12d-15d (c), 15d-21d (d), 21d-P6 (e), P48-h6 (f). Vertical axis shows GO terms while horizontal axis shows number of genes involved. Additional file 6: Figure S3. Partial GO enrichment in molecular functions. The figure shows partial gene enrichment for differentially expressed miRNAs in terms of molecular function during 6d-8d (a), 8d-12d (b), 12d-15d (c), 15d-21d (d), 21d-P6 (e), P48-h6 (f). Vertical axis shows GO terms while horizontal axis shows number of genes involved.
