# Supplementary material for: Identification of miRNAs during mouse postnatal ovarian development and superovulation
Source: J Ovarian Res. 2015 Jul 8;8:44. doi: 10.1186/s13048-015-0170-2 (PMC4499447; doi:10.1186/s13048-015-0170-2)
Supplement: Additional file 1: Table S1. — Survey of miRDeep2 performance for score cut-offs 0 to 10. [file 13048_2015_170_MOESM1_ESM.doc]

**Supplementary Table 1 Survey of miRDeep2 performance for score cut-offs 0 to 10.**

|  | **Novel miRNAs** | | | **Known miRBase miRNAs** | | |  |
| --- | --- | --- | --- | --- | --- | --- | --- |
| [miRDeep2 score](http://www.nature.com/nbt/journal/v26/n4/abs/nbt1394.html)^1^ | [Predicted by mirdeep2](../../../../E:%5CPhD%20stuff%5CDeep%20sequencing%5CmiRNA%20Data%5CData%2001%5C5_novel_miRNA_prediction%5CTable%204.1预测识别新miRNA分析结果.html)^2^ | [Estimated false positives](../../../../E:%5CPhD%20stuff%5CDeep%20sequencing%5CmiRNA%20Data%5CData%2001%5C5_novel_miRNA_prediction%5CTable%204.1预测识别新miRNA分析结果.html)^3^ | [Estimated true positives](../../../../E:%5CPhD%20stuff%5CDeep%20sequencing%5CmiRNA%20Data%5CData%2001%5C5_novel_miRNA_prediction%5CTable%204.1预测识别新miRNA分析结果.html)^4^ | [in species](../../../../E:%5CPhD%20stuff%5CDeep%20sequencing%5CmiRNA%20Data%5CData%2001%5C5_novel_miRNA_prediction%5CTable%204.1预测识别新miRNA分析结果.html)^5^ | [in data](../../../../E:%5CPhD%20stuff%5CDeep%20sequencing%5CmiRNA%20Data%5CData%2001%5C5_novel_miRNA_prediction%5CTable%204.1预测识别新miRNA分析结果.html)^6^ | [Detected by mirdeep2](../../../../E:%5CPhD%20stuff%5CDeep%20sequencing%5CmiRNA%20Data%5CData%2001%5C5_novel_miRNA_prediction%5CTable%204.1预测识别新miRNA分析结果.html)^7^ | [Estimated signal-to-noise](../../../../E:%5CPhD%20stuff%5CDeep%20sequencing%5CmiRNA%20Data%5CData%2001%5C5_novel_miRNA_prediction%5CTable%204.1预测识别新miRNA分析结果.html)^8^ |
| 10 | 86 | 20 ± 5 | 66 ± 5 (76 ± 5%) | 1157 | 937 | 532 (57%) | 13.8 |
| 9 | 93 | 21 ± 5 | 72 ± 5 (77 ± 5%) | 1157 | 937 | 534 (57%) | 13.6 |
| 8 | 98 | 22 ± 5 | 76 ± 5 (77 ± 5%) | 1157 | 937 | 536 (57%) | 13.4 |
| 7 | 107 | 23 ± 5 | 84 ± 5 (78 ± 5%) | 1157 | 937 | 539 (58%) | 13.2 |
| 6 | 113 | 25 ± 6 | 88 ± 6 (78 ± 5%) | 1157 | 937 | 543 (58%) | 12.6 |
| **5** | **160** | **31 ± 7** | **129 ± 7 (81 ± 4%)** | **1157** | **937** | **655 (70%)** | **12.1** |
| 4 | 196 | 55 ± 8 | 141 ± 8 (72 ± 4%) | 1157 | 937 | 686 (73%) | 7.7 |
| 3 | 229 | 170 ± 15 | 59 ± 15 (26 ± 6%) | 1157 | 937 | 697 (74%) | 3 |
| 2 | 267 | 247 ± 17 | 21 ± 15 (8 ± 6%) | 1157 | 937 | 711 (76%) | 2.3 |
| 1 | 410 | 326 ± 19 | 84 ± 19 (20 ± 5%) | 1157 | 937 | 752 (80%) | 2.2 |
| 0 | 535 | 500 ± 24 | 36 ± 23 (7 ± 4%) | 1157 | 937 | 759 (81%) | 1.7 |

^1^ miRDeep2 score signify the log-odds probability of a sequence being true miRNA precursor compared to probability that it is a background hairpin.

^2^Number of novel miRNA hairpins with a score ≥ cut-off value.

^3^Number of false positive miRNA hairpins predicted at this cut-off, as estimated by the miRDeep2 controls. Mean and standard deviation are estimated from 100 rounds of permuted controls.

^4^Number of true positive miRNA hairpins is estimated as t = total novel miRNAs - false positive novel miRNAs. The percentage of the predicted novel miRNAs that is estimated to be true positives is calculated as p = t/total novel miRNAs. The number of false positives is estimated from 100 rounds of permuted controls. In each of the 100 rounds, t and p are calculated, generating mean and standard deviation of t and p. The variable p can be used as an estimation of miRDeep2 positive predictive value at the score cut-off.

^5^Reference mature miRNAs for human given as input to miRDeep2.

^6^Number of reference mature miRNAs perfectly mapped to one or more of precursor candidates that have been excised from the genome by miRDeep2.

^7^Number of reference mature miRNAs perfectly mapped to one or more of predicted miRNA hairpins that have a score equal to or above the cut-off value. The percentage of reference mature miRNAs in data that is detected by miRDeep2 (s = reference mature miRNAs detected/reference mature miRNAs in data. s can be used as an estimation of miRDeep2 sensitivity at the score cut-off).

^8^The signal-to-noise ratio for the given score cut-off was computed as r = total miRNA hairpins reported / mean estimated false positive miRNA hairpins over 100 rounds of permuted controls.
