# Supplementary figures and images for: Identification of miRNAs during mouse postnatal ovarian development and superovulation
Source: J Ovarian Res. 2015 Jul 8;8:44. doi: 10.1186/s13048-015-0170-2 (PMC4499447; doi:10.1186/s13048-015-0170-2)

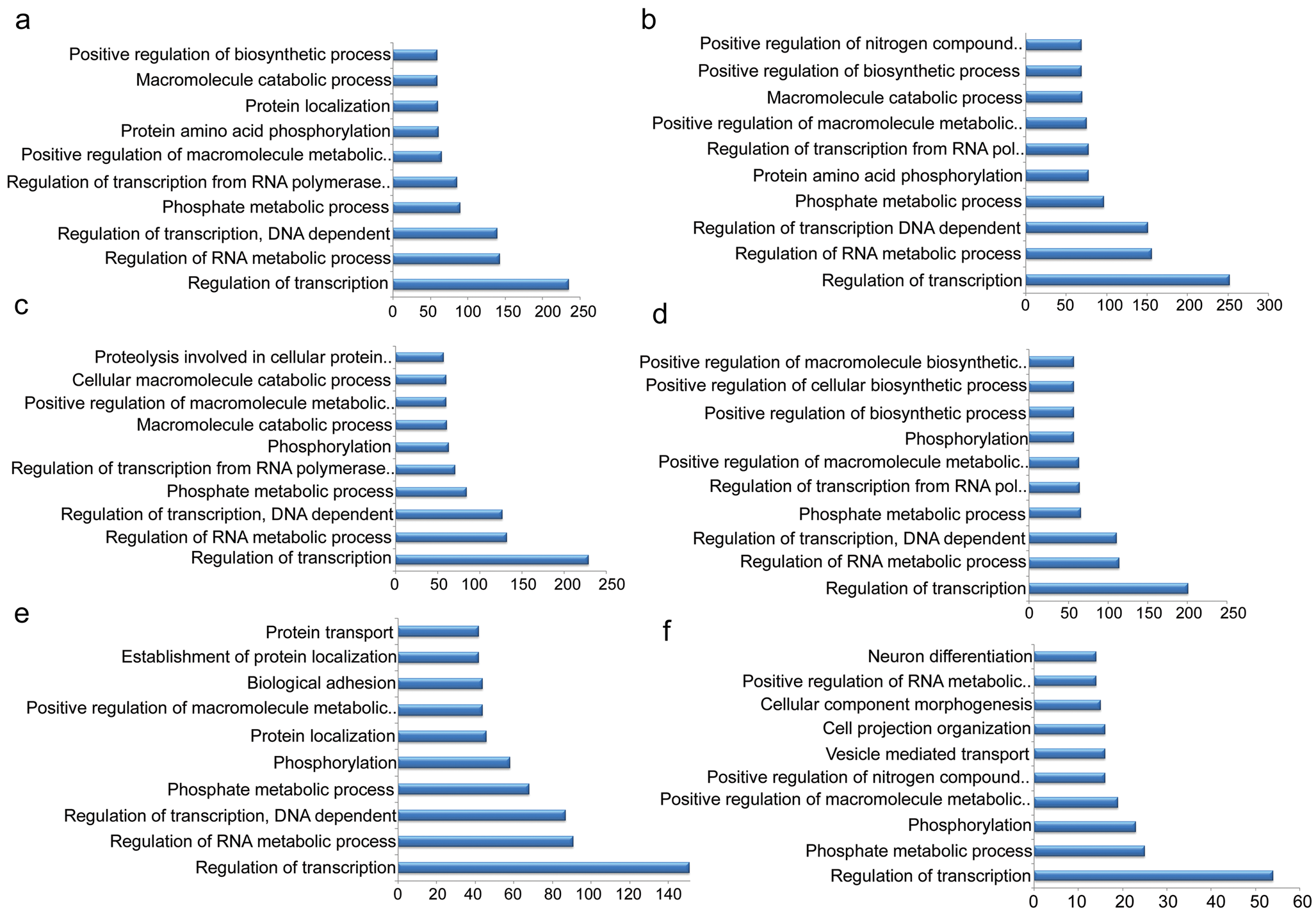

Supplement: Additional file 4: Figure S1. — Partial GO enrichment in biological process. The figure shows partial gene enrichment for differentially expressed miRNAs in terms of biological process during 6d-8d (a), 8d-12d (b), 12d-15d (c), 15d-21d (d), 21d-P6 (e), P48-h6 (f). Vertical axis shows GO terms while horizontal axis shows number of genes involved. [file 13048_2015_170_MOESM4_ESM.tif]

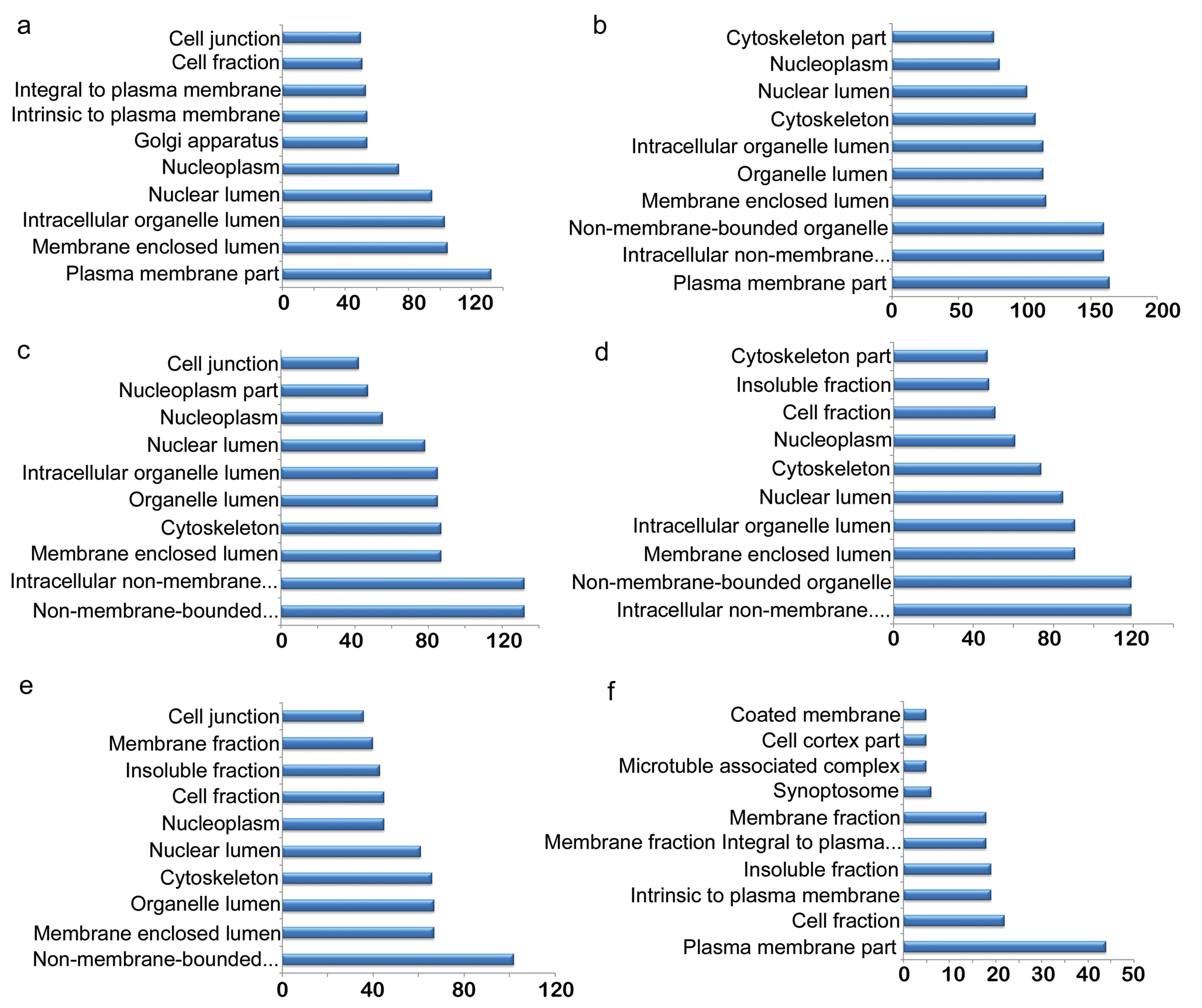

Supplement: Additional file 5: Figure S2. — Partial GO enrichment in cellular components. The figure shows partial gene enrichment for differentially expressed miRNAs in terms of cellular components during 6d-8d (a), 8d-12d (b), 12d-15d (c), 15d-21d (d), 21d-P6 (e), P48-h6 (f). Vertical axis shows GO terms while horizontal axis shows number of genes involved. [file 13048_2015_170_MOESM5_ESM.tif]

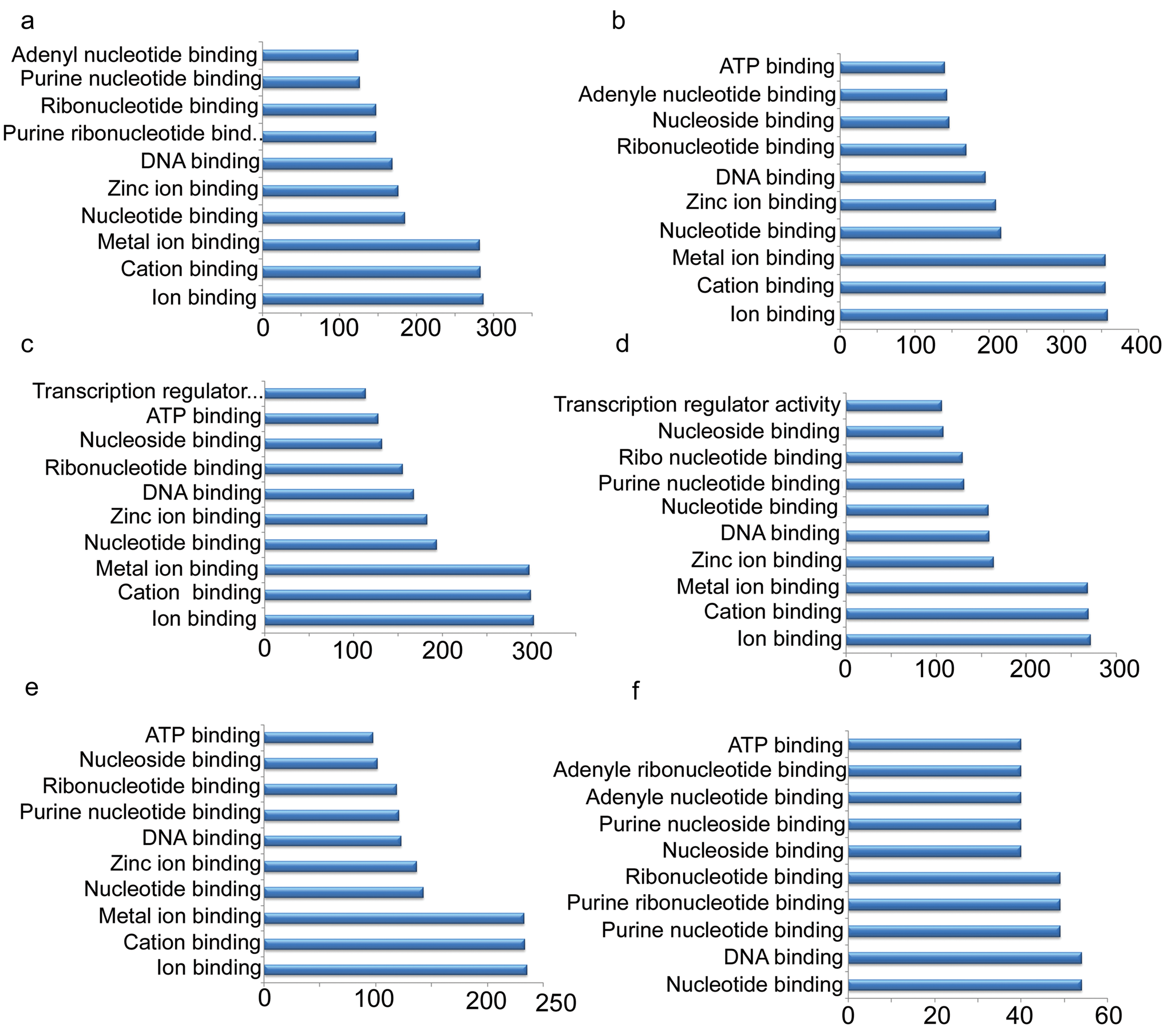

Supplement: Additional file 6: Figure S3. — Partial GO enrichment in molecular functions. The figure shows partial gene enrichment for differentially expressed miRNAs in terms of molecular function during 6d-8d (a), 8d-12d (b), 12d-15d (c), 15d-21d (d), 21d-P6 (e), P48-h6 (f). Vertical axis shows GO terms while horizontal axis shows number of genes involved. [file 13048_2015_170_MOESM6_ESM.tif]
